# Small antibody fusion proteins with complementarity-determining regions and lidamycin for tumor targeting therapy

**DOI:** 10.3892/ol.2013.1143

**Published:** 2013-01-18

**Authors:** GEN-SHEN ZHONG, MIN-NA WU, XIAO-FANG GUO, ZHI-SHAN XU, SHENG-HUA ZHANG, YONG-SU ZHEN

**Affiliations:** 1 The First Affiliated Hospital of Xinxiang Medical University, Xinxiang, Henan 453100;; 2 Xinxiang Medical University, Xinxiang, Henan 453003;; 3 Institute of Medicinal Biotechnology, Chinese Academy of Medical Sciences and Peking Union Medical College, Beijing 100050, P.R. China

**Keywords:** gelatinases, lidamycin, complementarity-determining region-3, tumor therapy

## Abstract

Gelatinases are overexpressed in several types of maligancies and tumor stromal cells. Lidamycin is an enediyne antitumor antibiotic, which is composed of an apoprotein (LDP) and an active chromophore (AE). It is known that the heavy-chain complementarity-determining region-3 (CDR3) domain of scFv is important in antibody affinity. The aim of this study was to prepare the enediyne-energized fusion proteins with a heavy-chain CDR3 domain of anti-gelatinases scFv and lidamycin, and to evaluate their antitumor efficiency. Fusion proteins comprising the CDR3 domain and the lidamycin apoprotein were generated, and ELISA, immunofluorescence and FACS were used to analyze the binding of the fusion protein with antigen gelatinases. The purified fusion proteins were assembled with the lidamycin chromophore, and the antitumor effects were evaluated *in vitro* and *in vivo*. It was found that the CDR3-LDP and CDR3-LDP-CDR3 fusion proteins demonstrated high affinity towards antigen gelatinases. Following stimulation of CDR3-LDP with enediyne, the results of MTT showed potent cytotoxicity towards tumor cells; the IC50 values of CDR3-LDP-AE to HepG2 and Bel-7402 tumor cells were 1.05×10^−11^ and 6.6×10^−14^ M, respectively. In addition, CDR3-LDP-AE displayed a potent antitumor effect in H22 cell xenografts in mice; the combination of CDR3-LDP (10 mg/kg) and CDR3-LDP-AE (0.25 and 0.5 mg/kg) revealed that the tumor inhibitory rates were 85.2 and 92.7%, respectively (P<0.05 compared with CDR3-LDP-AE). In conclusion, these results suggest that the CDR3-LDP fusion protein and its analog CDR3-LDP-AE may both be promising candidates for tumor targeting therapy.

## Introduction

Gelatinases, also named type IV collagenases, include gelatinase A (MMP-2) and gelatinase B (MMP-9) (1). It is known that gelatinases are important in tumor progression and are overexpressed in different types of tumor cells, thus representing important tumor-associated antigens (2,3). Solid tumors are usually composed mainly of tumor cells and partly of stromal cells; the latter consist of endothelial cells, neutrophil cells and hemopoietic progenitor cells, which comprise the significant tumor microenvironment and are important in tumor development and drug therapy (4). It has been demonstrated that gelatinases are also overexpressed in the tumor stromal cells (4,5). Therefore, it may be promising to develop new therapeutic drugs using gelatinases as a potential target, which could kill the tumor cells as well as imbalance the tumor microenvironment homeostasis.

Lidamycin is an extremely potent antitumor antibiotic, which is composed of a highly active enediyne chromophore and a protecting apoprotein (6). The chromophore and the apoprotein may be disconnected and reconstituted under certain conditions (6). Taking advantage of the specific targeting capability of antibody fragments, different types of fusion protein, which were composed of small antibody fragments and the lidamycin apoprotein, were created. Following stimulation with enediyne, the fusion protein demonstrated antitumor efficiency i*n vitro* and *in vivo*(6–10).

It is known that the heavy-chain complementarity-determining region-3 (CDR3) domain of scFv is important in antigen binding (11). Qiu *et al* demonstrated that the fusion of several different CDR3 domains led to a good targeting efficiency (12). Our previous studies also demonstrated that fusion proteins containing the LDP and oligopeptides specific for tumor antigens exhibited potent antitumor activities (7,13), which suggested that a fusion protein containing LDP and tumor specific oligopeptides was a promising agent for development. We suggested that the combination of the enediyne-energized fusion protein with its analog led to augmented antitumor efficiency *in vivo*(10); however, the high-dose intravenous administration of fusion protein may facilitate an increase in immunogenicity. The possibility that a human anti-mouse antibody (HAMA) response may be induced by the antibody of murine origin, and that the down-sized antibody may more easily penetrate the core of the solid tumor with lower immunogenicity were considered. In the present study, fusion proteins containing the lidamycin apoprotein and one or two heavy-chain CDR3 domains of anti-gelatinases scFv were created, and their biomedical characterization was investigated.

## Materials and methods

### Construcion of recombiant plasmids pET-CDR3-LDP and pET-CDR3-LDP-CDR3

**Figure 1:**
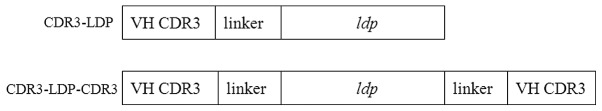
Diagram of NdeI/Xho I gene fragments encoded for the CDR3-LDP and CDR3-LDP-CDR3 fusion proteins. The gene sequence of VH CDR3 was TGTGCTAGAGGGGACTACTATAGGCGCTACTTTGAC. A linker of (G4S)2 was placed between VH CDR3 and the *ldp* domains.

The sequence of the heavy chain CDR3 domain of antigelatinase scFv (GenBank No. FJ037775) was TGTGCTAGAGGGGACTACTATAGGCGCTACTTTGAC. To construct the CDR3-LDP fusion protein, (GGGGS)2 was inserted betweeen the LDP and CDR3 domains as a linker (Fig. 1). Two primers was designed and the sequences used were as follows: P1: 5′-GGAATTCCATATGTGTGCTAGA GGGGACTACTATAGGCGCTACTTTGACGGTGGAGGT GGTTCAGGTGGA-3′ (the *Nde*I site is underlined) and P2: 5′-CCGCTCGAGGCCGAAGGTCAGAGCCACGTG-3′ (the *Xho*I site is underlined). Using the plasmid pET-Ec-ldp-Hr (7) as a template, and primers P1 and P2, PCR amplification was performed. The obtained fragment was *Nde*I/*Xho*I digested and was inserted into a pET30a(+) expression vector to generate the recombinant plasmid pET-CDR3-LDP. DNA sequencing analysis was conducted to verify that the gene sequence was correct (Invitrogen Life Technologies, Carlsbad, CA, USA).

To construct the pET-CDR3-LDP-CDR3 recombinant plasmid, three different primer were designed and the sequences used were as follows: P3: 5′-CCTTGCC GAAGATCCTCCACCTCCAGATCCTCCCCCGCCGCCG AAGGTCAGACCAC-3′; P4: 5′-CCGCTCGAGATCGAAAT ATCGTCTGATAATCTCCCCTTGCCGAAGATCCTCC-3′; P5: 5′-GGAATTCCATATGTGTGCT-3′. Using the pETEc-ldp-Hr as a template, and primers P1 and P3, PCR amplification was conducted. The amplified product was used as the next template, and P4 and P5 as the primers in the second PCR amplification. The final product was *Nde*I/*Xho*I digested and inserted into to the pET30a(+) plasmid to generate the recombinant plasmid pET-cdr3-ldp-cdr3. Additionally, the sequence analysis of pET-cdr3-ldp-cdr3 was verified (Invitrogen Life Technologies).

### Expression and purification of CDR3-LDP and CDR3-LDP- CDR3

The sequence-verified plasmids pET-CDR3-LDP and pET-CDR3-LDP-CDR3 were transformed into the *E.coli* BL21 (DE3) expression strain (Novagen/MerckKGaA, Darmstadt, Germany) to produce the recombinant protein. Expression, purification of CDR3-LDP and CDR3-LDP-CDR3 fusion protein was carried out according to the manufacturer’s protocol (Novagen). The purified protein was analyzed by SDS-PAGE and the protein concentration was determined by the BCA kit (Pierce Biotechnology, Inc., Rockford, IL, USA).

### Binding with gelatinases

Gelatinases were coated in a 96-well plate overnight, and a serial dilution of purified fusion proteins CDR3-LDP and CDR3-LDP-CDR3 was added. The detailed procedure was described previously (14), and the final affintiy constant was determined by Graphpad Prism 5 software (San Diego, CA, USA).

### Binding activities of fusion protein CDR3-LDP with tumor cells

Binding with tumor cells was determined by ELISA assay. Human Bel-7402 and HepG2 hepatoma cell lines were seeded in 96-well plate at a density of 1×10^4^cells/well and cultivated overnight at 37°C. The following procedure was performed according to that of our previous study (14).

To further identify the binding affinity of fusion protein to target tumor cells, we used a fluorescence-activated cell sorting (FACS)-based analysis assay. Protein bovine serum albumin (BSA), LDP and CDR3-LDP were FITC labeled for 16 h in a carbonate buffer solution (100 mmol/l NaHCO_3_; 10 mmol/l Na_2_CO_3_, pH 9.0) at 4°C. Labeled protein was separated from unbound FITC using the Sephadex G-25 column (GE Healthcare, Waukesha, WI, USA). Each FITC-labeled protein, BSA, LDP and CDR3-LDP, were incubated with 5×10^5^ Bel-7402 and HepG2 cells in a 100-μl volume of FACS buffer (PBS with 2% fetal bovine serum) for 2 h at room temperature. Following three washes with 500 μl of FACS buffer, cells were analyzed with a BD FACSCalibur (BD Biosciences San Jose, CA, USA).

Additionally, the binding specificity of CDR3-LDP with cancer cells was assessed by immunofluorescence. HepG2 cells (1×10^5^) were grown on coverslides overnight, fixed with ice-cold 70% methanol, blocked with 5% BSA, then incubated with CDR3-LDP fusion protein (100 μg/ml) for 2 h at 37°C. After washing with PBS, cells were incubated with mouse anti-His tag monoclonal antibody (dilution 1:200; Novagen) for 1 h, followed with FITC-conjugated goat anti-mouse antibody (dilution 1:500; Zhongshan Golden Bridge Biotechnology, Beijing, China). The images were observed under a fluorescence microscope and collected by fluorescence microscopy (Nikon TE 2000u, Tokyo, Japan).

### Preparation of enediyne-energized fusion protein CDR3-LDP-AE

To establish the potent antitumor activity of fusion protein CDR3-LDP, assembly of the fusion protein with the enediyne chromophore was performed. The detailed procedures and the HPLC analysis were all performed according to our previous study (10).

### MTT assay

The MTT assay was used for measuring *in vitro* cytotoxicity of stimulated CDR3-LDP-AE fusion protein as described previously (10). Cells were seeded at 3,000 cells/well in 96-well plates and incubated in 37°C for overnight. Subsequently, cells were exposed to different concentrations of lidamycin and stimulated CDR3-LDP-AE fusion protein for 48 h. MTT (Sigma, St. Louis, MO, USA) solution (5 mg/ml, 20 μl) was added to each well and incubated for a further 4 h at 37°C. The supernatant was removed and 150 μl DMSO was added to each well. The absorbance at 570 nm was measured using an ELISA reader (Thermo Fisher Scientific). Growth inhibition was calculated as a percentage of the nontreated controls.

### In vivo antitumor activity

The *in vivo* experiment was performed with 7-week-old female Kunming mice (KM), which were purchased from the Institute of Animal Research, Chinese Academy of Medical Science. The study protocols were according to the regulations of the Good Laboratory Practice for non-clinical laboratory studies of drugs issued by the National Scientific and Technologic Committee of People’s Republic of China.

Hepatoma 22 (H22) cells suspended in sterile saline were inoculated subcutaneously (at day 0) in the right axilla of mice, at a density of 2.0×10^6^ cells/0.2 ml/mouse. The mice were divided into seven groups, with 10 mice in each group. At 24 h of H22 cell transplantation (day 1), CDR3-LDP-AE was administered at doses of 0.25 and 0.5 mg/kg of body weight. lidamycin and CDR3-LDP fusion proteins were administered at doses of 0.06 and 10 mg/kg, respectively. The combination of CDR3-LDP (10 mg/kg) fusion protein with CDR3-LDP-AE (0.25 and 0.5 mg/kg) was also administered. All treatments were administered by intravenous injection into the tail vein in 200 ml of sterile saline solution. The experiment was terminated at day 11, and the tumors were excised and weighed. The mean tumor weight was calculated and the results were expressed as the mean ± standard deviation. The tumor inhibition rate was calculated by: 1 - Tumor weight (treated) / tumor weight (control) × 100.

### Statistical methods

Results of quantitative data in this study were presented as the mean ± standard deviation. Significant differences between two values were determined using the Student’s t-test. P<0.05 was considered to indicate a statistically significant difference.

## Results

### Expression and purification of CDR3-LDP and CDR3-LDP-CDR3 fusion proteins

**Figure 2:**
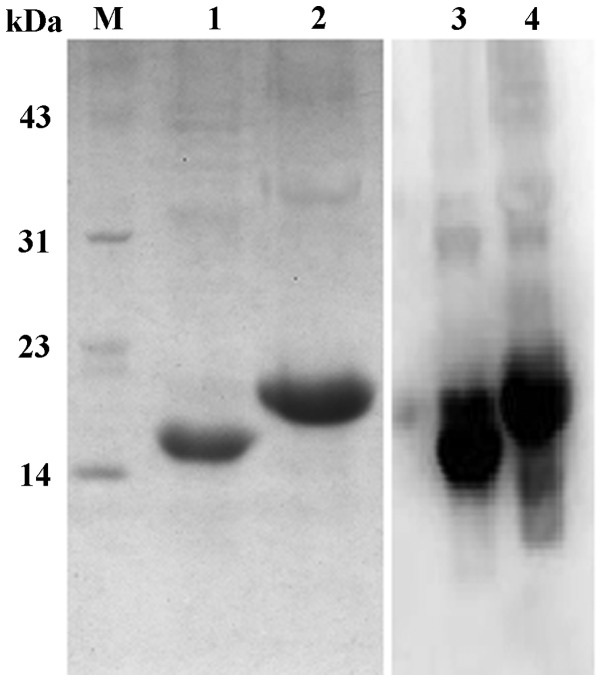
SDS-PAGE and western blot analysis of CDR3-LDP and CDR3-LDP-CDR3 fusion proteins following purification. M, marker; lanes 1 and 2, CDR3-LDP and CDR3-LDP-CDR3 fusion proteins, respectively; lanes 3 and 4, western-blot analysis of CDR3-LDP and CDR3-LDP-CDR3 using anti-His tag antibody, respectively.

As shown in Fig. 1, the recombinant plasmids pET-CDR3-LDP and pET-CDR3-LDPCDR3 were constructed. The plasmid were transferred to *E.coli* BL21 (DE3) for expression. Following cultivation for 4 h at 30°C and induction by 0.2 mM IPTG for an additional 3 h, the cultures were collected and washed with Tris-HCl buffer. Cultures were then treated with ultrasound to break down the cells. The supernatant was collected and loaded onto an Ni-affinity column for purification. SDS-PAGE was used to analyze the purified CDR3-LDP and CDR3-LDP-CDR3 fusion proteins. As shown in Fig. 2, the CDR3-LDP fusion protein migrated at a molecular weight of 15000 Da, while that of CDR3-LDP-CDR3 was 18,000 Da. These findings were in accordance with the theoretical prediction. Western blot analysis using anti-His tag antibody confirmed that the (His)_6_ tag was introduced into the fusion protein successfully.

### Affinity assay

**Figure 3:**
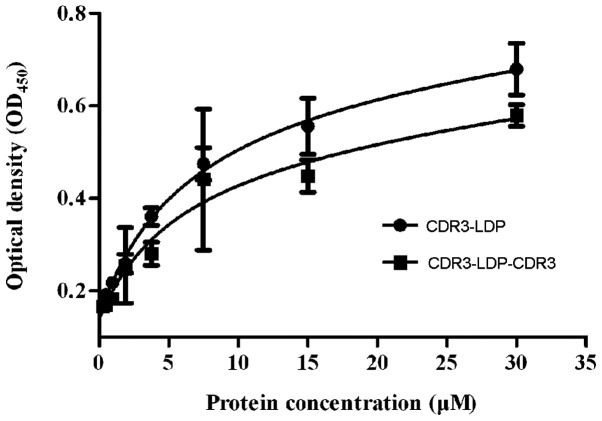
Binding affinity of CDR3-LDP and CDR3-LDP-CDR3 was determined by ELISA. The 96-well plate was pre-coated with gelatinases and incubated with increasing concentrations of each fusion protein and then with anti-His tag antibody. Data are presented as mean ± standard deviation, and were analyzed with Graphpad Prism 5 software.

ELISA was used to determine the binding of the CDR3-LDP and CDR3-LDP-CDR3 fusion proteins with antigen gelatinases. As shown in Fig. 3, no difference in affinity activity between fusion proteins CDR3-LDP and CDR3-LDP-CDR3 with gelatinases was observed; their affinity constant Kd values were 5.78×10^−6^ and 6.563×10^−6^ M, respectively. By contrast, the binding affinity of the CDR3-LDP fusion protein with gelatinases was relatively higher compared with that of the CDR3-LDP-CDR3 protein. The reason for this may be that the two CDR3 domains did not have a synergistic effect on binding with gelatinases, and the fused (His)_6_ tag may also have had a negative impact on antigen binding, thus resulting in that the binding activity of CDR3-LDP-CDR3 did not exhibit a higher affinity compared with that of CDR3-LDP. As the volu-metric productivity of the CDR3-LDP-CDR3 fusion protein expressed in *E.coli* in a soluble form was lower compared with that of CDR3-LDP and did not demonstrate enhanced binding activity with gelatinases, the CDR3-LDP fusion protein was chosen for the following investigation.

### Immunofluorescence assay

**Figure 4:**
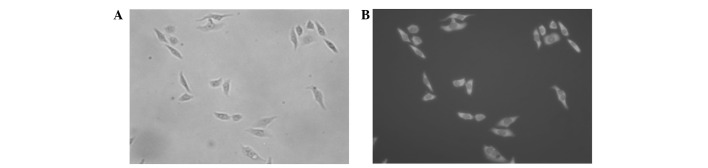
The immunofluorescence of fusion protein CDR3-LDP with HepG2 tumor cell lines. (A) was observed with phase contrast and (B) with immunofluorescence microscopy.

Immunofluorescence was used to investigate the binding specifity of the CDR3-LDP fusion protein with HepG2 cells *in vitro*. As shown in Fig. 4, the CDR3-LDP fusion protein was able to bind well with HepG2 cells, indicating their gelatinases were abundantly expressed around the cell membrane.

### Flow cytometry analysis of CDR3-LDP with tumor cells

**Figure 5:**
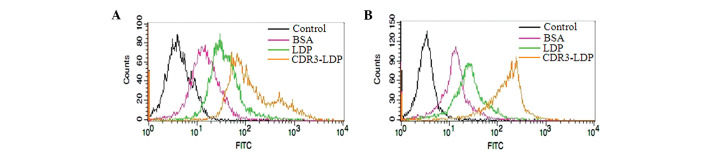
Flow cytometric results of the comparison of binding capability with Bel-7402 (A) and HepG2 (B) tumor cell lines between BSA, LDP and CDR3-LDP. Autofluorescence measurements are in black (negative control); the rose-bengal curve represents cells cultured with BSA; the green curve represents cells cultured with LDP and the orange curve depicts the binding ability of CDR3-LDP with cell lines.

Flow cytometry was conducted to further testify that the attachment of the CDR3-LDP domain was capable of improving the binding of LDP with tumor cells. As shown in Fig. 5, compared with the FITC-labeled BSA and LDP protein, the FITC-labeled CDR3-LDP was able to enhance the binding intensity with tumor cells; the attachment of a single CDR3 domain increased the binding of LDP with tumor cells.

### The assembly of CDR3-LDP fusion protein with lidamycin AE

**Figure 6:**
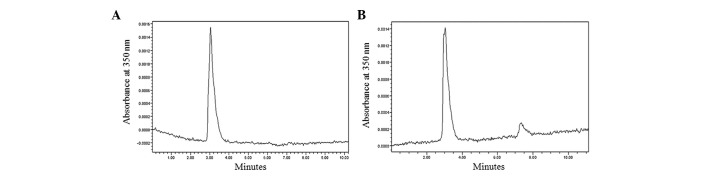
HPLC analysis of fusion protein CDR3-LDP-AE. The chromatography of CDR3-LDP fusion protein (A) and enediyne-energized CDR3-LDP-AE fusion protein (B). The monitored wavelength was 280 nm. The small peak indicates the peak of chromophore AE.

To establish the potent antitumor activity of CDR3-LDP, the assembly procedure was performed. As shown in Fig. 6A, the RP-HPLC chromatogram indicated that the purity of purified CDR3-LDP was >90%, which satisfied the requirements of the experiment. Following AE assembly, the enediyne-energized CDR3-LDP-AE fusion protein was obtained. As shown in Fig. 6B, an additional peak was evident in the chromatogram and the retention time was ∼7.3 min, which was similar to that of our previous study (10).

### MTT assay

**Figure 7:**
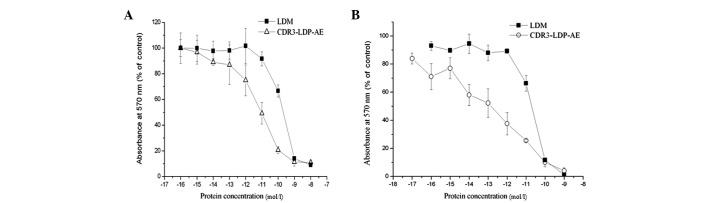
The cytotoxicity of energized fusion protein CDR3-LDP-AE on hepatoma Bel-7402 (A) and HepG2 (B) cancer cells as determined by MTT assay.

The MTT assay was used to determined enediyneenergized CDR3-LDP-AE fusion protein cytotoxicity to Bel-7402 and HepG2 cells. As shown in Fig. 7, CDR3-LDP demonstrated extremely potent cytotoxicity to tumor cells; the IC50 values for Bel-7402 and HepG2 cells were 1.05×10^−11^ and 6.6×10^−14^ M, respectively. By contrast, the IC50 values of lidamycin to Bel-7402 and HepG2 cells were 5.6×10^−10^ and 4.2×10^−11^ M, respectively. The enhancement in cytotoxicity of CDR3-LDP-AE may be related to the increased binding capability with tumor cells compared with that of lidamycin.

### In vivo animal experiment

To evaluate the antitumor efficiency of CDR3-LDP-AE *in vivo* and to further demonstrate that the combined therapy of fusion protein with enediyne-energized fusion protein augmented the antitumor effect *in vivo* as observed in our previous study (10), the H22 cell xenografts in mice were used to evaluate the antitumor efficiency of fusion proteins CDR3-LDP and CDR3-LDP-AE, both alone and in combination.

As shown in Table I, 10 mg/kg CDR3-LDP had an inhibition rate of 50%, which indicated that the fusion protein alone exerted certain antitumor effects. Following assembly with AE, 0.25 and 0.5 mg/kg enediyne-energized CDR3-LDP-AE fusion protein demonstrated tumor inhibition rates of 78.8 and 87.1%, respectively (P<0.05 compared with those of CDR3-LDP and lidamycin). This indicated that the assembly of AE improved the antitumor efficacy of the CDR3-LDP fusion protein, and also suggested that the attachment of CDR3 domain increased the accumulation of lidamycin at the tumor site. The combination of CDR3-LDP (10 mg/kg) and CDR3-LDP-AE (0.25 and 0.5 mg/kg) further augmented the tumor inhibitory efficiency; the tumor inhibition rates were increased to 85.2 and 92.7%, respectively (P<0.05 compared with CDR3-LDP-AE). The increase in antitumor efficacy was not accompanied with a loss of body weight, which indicated that the combined therapy was effective and further improved the antitumor efficacy.

## Discussion

Lidamycin is a potent antitumor antibiotic, which consisted of a protecting apoprotein and a highly active enediyne chromophore. The apoprotein and the chromophore may be detached and reassembled under certain conditions (6,15). Taking advantage of the targeting property of antibodies or antibody fragments, types of chimeric fusion proteins, composed of antibody fragments and apoprotein, were created and then reassembled with the detached chromophore (6–9). In the present study, two fusion proteins CDR3-LDP and CDR3-LDP-CDR3, composed of a heavy-chain CDR3 domain fused with the lidamycin apoprotein, were designed. The aim of the study was to develop a targeting fusion protein with a lowered immunogenicity. As observed, both CDR3-LDP and CDR3-LDP-CDR3 were easily expressed in *E.coli*, and mainly existed in the soluble form. However, the volumetric productivity of CDR3-LDP-CDR3 was relatively lower compared with that of CDR3-LDP, and was easy to deposit, which may have been due to the addition of a further CDR3 domain and thus the increase in hydrophobicity. The results of the ELISA suggested that there was no significant difference in the binding activity between CDR3-LDP and CDR3-LDP-CDR3 fusion proteins. The addition of a further CDR3 domain failed to increase the binding activities. Therefore, for the development of this type of lidamycin fusion protein, it is necessary to consider the negative influences of steric hindrance or the existence of His tag on the affinity property of oligopeptide. However, the ELISA is relatively accurate approach to determine the affinity activity, which may be determined by surface plasmon resonance (SPR) or other more accurate equipment (16,17). As the CDR3-LDP-CDR3 fusion protein did not demonstrate improved binding activity, the CDR3-LDP fusion protein was chosen to perform the following investigations.

The results of ELISA and immunofluorescence demonstrated that the CDR3-LDP fusion protein could bind well with the tumor cells, and the results of FACS further confirmed that the CDR3 domain increased the binding of LDP with tumor cells. Following AE assembly, the enediyne-energized CDR3-LDP-AE fusion protein displayed more potent cytotoxicity activity to tumor cells compared with lidamycin, this may have been due to the increased binding capability of CDR3-LDP-AE with tumor cells, as the protein was fused with a tumor-binding oligopeptide CDR3 domain. Notably, in the mouse model for the transplantation of H22 cells, 10 mg/kg CDR3-LDP fusion protein alone had moderate antitumor efficiency, and this was similar to the *in vivo* result of apoprotein produced by genetic engineering (18). The lidamycin apoprotein does not merely act as a carrier of chromophore; its antitumor mechanism requires further elucidation. Following the assembly of the chromophore with CDR3-LDP, the assembly product CDR3-LDP-AE demonstrated enhanced antitumor efficiency. Additionally, the combination of a large concentration of fusion protein CDR3-LDP and a small concentration of enediyne-energized CDR3-LDP-AE fusion protein further augmented the antitumor effect, indicating that the combined therapeutic strategy was effective and has further potential for the development of fusion protein containing lidamycin apoprotein. This strategy would enhance the antitumor effect without being accompanied with an increase in side effects. As the molecular weight of CDR3-LDP was ∼15000 Da, the immunogenicity of CDR3-LDP may be greatly lower compared with that of dFv-LDP-AE as demonstrated previously (10). However, the binding activity of CDR3-LDP was ∼100-fold lower compared with that of dFv-LDP. Although the higher affinity does not negate superior tumor targeting capability (12), it has been demonstrated that the higher the affinity, the lower the tumor penetrating ability (19,20). Therefore, in the development of a lidamycin fusion protein, our long-term aim was to design a tumor-targeting peptide or antibody fragment, and select the fusion protein with lower immunogenicity, and stronger tumor targeting and penetrating properties for further development. Our efforts should also be directed toward the development of a novel administrative approach or the humanization of antibodies.

## Figures and Tables

**Table I:** The tumor growth inhibition effects of fusion protein CDR3-LDP, CDR3-LDP-AE and their combination on hepatoma 22 cells in mice.

Groups	Dosage (mg/kg)	Mouse number (begin/end)	BWC (g)	Tumor weight (g)	Inhibition ratio (%)
Control		10/10	+5.72	1.401±0.234	-
LDM	0.06	10/10	+4.09	0.513±0.281	63.4
CDR3-LDP	10.0	10/10	+4.69	0.613±0.523	56.2
CDR3-LDP-AE	0.25	10/10	+2.43	0.297±0.137	78.8^ab^
	0.5	10/10	−0.47	0.180±0.091	87.1^ab^
CDR3-LDP	10+0.25	10/10	+2.15	0.208±0.067	85.2^c^
+ CDR3-LDP-AE	10+0.5	10/10	−0.16	0.103±0.037	92.7^c^

a Compared with LDM, P<0.05;

b compared with fusion protein CDR3-LDP, P<0.05;

c compared with CDR3-LDP-AE (0.25 and 0.5 mg/kg), P<0.05. BWC, body weight change. LDM, lidamycin.
